# From genetics and cerebral asymmetry, through motor dysfunction intrinsic to psychosis, to early intervention: elaborating the seminal contributions of Timothy J. Crow

**DOI:** 10.1017/S0033291725001254

**Published:** 2025-05-09

**Authors:** John L. Waddington

**Affiliations:** 1 School of Pharmacy and Biomolecular Sciences, RCSI University of Medicine and Health Sciences, Dublin, Ireland; 2 Jiangsu Key Laboratory of Translational Research and Therapy for Neuropsychiatric Disorders and Department of Pharmacology, College of Pharmaceutical Sciences, Soochow University, Suzhou, China

**Keywords:** Schizophrenia, psychosis, bipolar disorder, cerebral asymmetry, genetics, dysmorphogenesis, facial asymmetry, motor dysfunction, early intervention, duration of untreated psychosis (DUP)

Across six decades, few investigators and theoreticians have been so enduringly impactful across so many conceptual domains of schizophrenia and psychotic illness as Tim Crow. Following his recent death, Palaniyappan and Liddle (2025) present in their article ‘Seminal contributions of Timothy J. Crow’ a timely and insightful account of his life and work in terms of five conceptual domains with which he was associated. Palaniyappan and Liddle rightly note that his most intellectually profound work on genetics, cerebral asymmetry, language, and speciation was unfinished in his lifetime and that recent observations appear to diminish some of the premises of his arguments in this particular domain. Yet, other recent observations resonate with and elaborate important aspects of Crow’s arguments: polygenic risk scores for schizophrenia from genome-wide association studies (GWAS) are associated with neuroimaging indices of brain asymmetries in regions important for language and executive functions (Sha, Schijven, & Francks, 2021); additionally, a set of those genes associated with risk for schizophrenia by GWAS is associated with a variant index of loss of asymmetry in frontal brain regions in this disorder (Sukno et al., 2024). Furthermore, two additional conceptual domains now constitute ‘received wisdom’ in which Crow’s pioneering insights have been substantially overlooked.

Regarding genetics and cerebral asymmetry, Crow’s work involves a fundamental challenge in vertebrate developmental biology that long predates his interest: what is the nature of initial establishment and subsequent breaking of symmetry during embryonic morphogenesis to create asymmetry? During early fetal life, what are to become the forebrain and the frontonasal prominences of the face develop in exquisite embryological intimacy, hence both acquire asymmetries (Rhodes, 2006; Schijven et al., 2023; Sha et al., 2021; Sukno et al., 2024). Indeed, normal facial asymmetry has long been recognized to be of considerable evolutionary and psychosocial import: greater facial symmetry is reliably deemed to be more attractive to conspecifics, with attendant implications for fecundity (Rhodes, 2006). Recently, it has been shown by Sukno et al. (2024) that: (i) the geometry of normal asymmetries in the upper facial region is similar to the geometry of normal asymmetries in frontal regions of the brain and (ii) the extent of such facial asymmetries in control subjects is notably reduced in schizophrenia. Therefore, in terms of both geometry and embryology, reduced upper facial asymmetries in schizophrenia are indicative of reduced forebrain asymmetries that relate to Crow’s arguments but have remained controversial (Schijven et al., 2023). Crow also concerned himself with the paradox of the enduring presence of schizophrenia in human populations despite this disorder being associated with reduced fecundity. In terms of speciation, one might expect this reduction in upper facial asymmetries in schizophrenia, with an attendant increase in attractiveness, to be associated with increased fecundity; thus, greater attractiveness associated with reduced upper facial asymmetry in schizophrenia may function to offset reduced fecundity.

These findings have been given import by GWAS, as detailed by Sukno et al. (2024): in healthy subjects, among genes associated with the geometry of both facial and cortical brain shape, 13 are also associated with risk for schizophrenia; among genes associated with the geometry of brain asymmetry, seven are also associated with risk for schizophrenia. Thus, upper facial asymmetries appear related to frontal lobe asymmetries in terms of geometry, embryology, and regulation by genes associated with risk for schizophrenia. That loss of these facial asymmetries is evident also in bipolar disorder would be in accordance with Crow’s arguments on the continuum of psychosis. While these recent findings, like those of Sha et al. (2021), do not map across Crow’s theorizing on a specific genetic basis for disrupted cerebral asymmetry in psychosis, they support several aspects of his arguments and suggest an unexpected connection with contemporary GWAS findings.

Regarding motor dysfunction in schizophrenia, colleagues in Crow’s group studied a rare but critical population of subjects with long-standing schizophrenia who had remained untreated with antipsychotic drugs. These subjects showed involuntary movement disorder that differed little in either quality or quantity from otherwise similar subjects who had received conventional long-term treatment with antipsychotic drugs; thus, Crow argued ‘It appears incontrovertible that a syndrome of abnormal involuntary movements, which has often been described as tardive dyskinesia and attributed to antipsychotic drug administration, occurs sometimes as a manifestation of the processes of schizophrenia and perhaps other diseases’ (Crow et al., 1982). While motor dysfunction intrinsic to psychotic disorders is now ‘received wisdom’ (Walther et al., 2020), the majority of studies make no reference to Crow’s prescience. Furthermore, this prescience is now reflected in contemporary models for the pathobiology of psychosis, which propose dysfunction in a corticostriatal–thalamocortical network that involves three parallel and overlapping corticostriatal pathways consisting of limbic, associative, and sensorimotor subdivisions (McCutcheon, Abi-Dargham, & Howes, 2019). Such network dysfunction indicates a substrate for the confluence of psychopathology and motor dysfunction that Crow argued to be intrinsic to psychotic illness.

In addition to these now six conceptual domains of schizophrenia and psychotic illness impacted by Crow, a seventh should be emphasized. His group initiated one of the earliest systematic studies of first-episode psychosis that included assessment of duration of untreated psychosis (DUP) and response to antipsychotics. On the basis of its findings, he considered whether ‘delay in institution of the treatment itself leads to poorer long-term outcome, that is that persistence of symptoms untreated by neuroleptic drugs leads to abnormality, which cannot be completely reversed by subsequent treatment’ and proposed that ‘the new issue raised by this study is whether medication instituted early and continued during and perhaps after an acute episode of illness will have enduring beneficial effects on the course of the condition’ (Crow, MacMillan, Johnson, & Johnstone, 1986). Here is a fully conceptualized model for the consequences of untreated psychosis and the need for early treatment to ameliorate the subsequent trajectory of the disorder, long prior to widespread advocacy and adoption of early intervention for psychosis. Such breadth and depth of prescience, intellectual rigor, and practical import in relation to the real-world challenges of psychotic illness is testamentary.
